# Knowledge and practices on dog bite management for rabies prevention in eThekwini, South Africa

**DOI:** 10.4102/jphia.v16i4.1391

**Published:** 2025-10-07

**Authors:** Khuliso Ravhuhali, Masingita Makamu, Sharika Naidoo, Sanele Zuma, Samkelisiwe Mvelase, Thuleleni Ntuli, Xolani Shandu, Vusani Myeni, Zinhle Buthelezi, Siphumelele Mlambo, Pumeza Hlanganyana, Siyabonga Mbanjwa, Jessica Thompson-Pillay, Sathee Devi Rambally, Muzi Phoswa, Sbusiso Mchunu, Ntobeko Zondi, Radiya Gangat, Poncho Phafane, Thembekile Zwane, Hellen Netshivhumbe, Emelda Ramutshila, Maxwell Mabona, Velile Ngidi, Leigh Johnston, Babongile Mhlongo, Lazarus Kuonza

**Affiliations:** 1 South Africa Field Epidemiology Training Programme, National Institute for Communicable Diseases, Johannesburg, South Africa; 2 Division of Public Health Surveillance and Response, National Institute for Communicable Diseases, Johannesburg, South Africa; 3 Department of Health, KwaZulu-Natal Province, Pietermaritzburg, South Africa; 4 Department of Communicable Diseases, eThekwini Municipality, Durban, South Africa; 5 Department of Environmental Health, eThekwini Municipality, Durban, South Africa; 6 Centre for Emerging, Zoonotic and Parasitic Diseases, National Institute for Communicable Diseases, Johannesburg, South Africa; 7 DG Murray Trust, Pietermaritzburg, South Africa; 8 National Institute for Communicable Diseases, Johannesburg, South Africa

**Keywords:** dog bite, human rabies, knowledge, attitude, practices, KwaZulu-Natal Province

## Abstract

**Background:**

Dogs are responsible for rabies virus transmission to humans in up to 99% of cases. Human rabies cases in the eThekwini district have led to human fatalities.

**Aim:**

The aim of this study is to evaluate the knowledge, attitudes and practices (KAP) of household heads (HHs) regarding the management of dog bites in the context of rabies prevention, along with the factors associated with these practices.

**Setting:**

The study was conducted in facility catchment areas that reported > 10 dog bite cases in 2023 in the South region of the eThekwini district, KwaZulu-Natal province.

**Methods:**

This was a cross-sectional survey, including HHs (≥ 18 years). An interviewer-administered questionnaire was used to collect data. Multivariable logistic regression was used to identify associated factors.

**Results:**

A total of 437 HHs were interviewed, including 258 (59%) females. The mean age was 40.6 (standard deviation [s.d.]: 15.7) years. Overall, 411 (94%) (95% confidence interval [CI]: 91.4% – 95.9%] of HHs had poor knowledge, 434 (99%) (95% CI: 98% – 99.8%) showed positive attitudes towards dog bite management and 102 (61%) (95% CI: 55.8% – 64.5%) had poor practices. In multivariable analysis, being aged 31–50 years (adjusted odds ratio [aOR] = 4.1; 95% CI: 0.86–19.3; *p* = 0.035) and having secondary education (aOR = 0.39; 95% CI: 0.17–0.92; *p* = 0.031) were associated with good knowledge. Owning a dog (aOR = 17.51; 95% CI: 10.3–29.6, *p* < 0.001) was associated with good practices towards dog bite management.

**Conclusion:**

It is recommended that the District Ministry of Health enhance public awareness on proper dog bite management and emphasise the importance of dog vaccination.

**Contribution:**

The study highlights inadequate knowledge and poor practices related to dog bite management in rabies prevention. Identifying these gaps is essential for developing targeted health education initiatives, which can support the global objective of eliminating human rabies deaths by 2030.

## Introduction

Dog bites can cause short- or long-term physical problems, such as injuries, infections, psychological traumas and even death, and they are also the primary means of transmitting the rabies virus to humans, accounting for up to 99% of cases.^1^ Rabies is a viral zoonotic disease that causes progressive and fatal inflammation of the brain and spinal cord.^1^ Human deaths from rabies are significantly underreported in many parts of the world. However, in over 150 countries, rabies is estimated to cause 59 000 deaths annually, with 95% of cases occurring in Africa and Asia.^2^ Approximately half of rabies cases occur in children under 15 years of age from rural populations.^2^ Rabies can affect both domestic and wild animals. However, domestic dogs transmit the rabies virus to humans in almost 99% of the cases.^2^ Between January 2023 and December 2023, South Africa recorded 10 cases of human rabies, and 5 of the cases were reported in the KwaZulu-Natal province.^3^ Rabies is a category one notifiable medical condition (NMC) in South Africa and needs to be reported immediately or within 24 h of diagnosis.^3^

Most communities know that rabies is transmitted through dog bites, with awareness rates ranging from 73% to over 95%.^3,4^ Knowledge about proper wound management post-dog bite is generally low. Only 5% in Tanzania and 7% in Ethiopia knew about prompt wound cleansing.^3,4^ In Bangladesh, 59% of victims first seek treatment from traditional healers, and only 29% receive the rabies vaccine.^5^

Rabies can be preventable through adequate vaccination of domestic dogs and cats and the use of rabies post-exposure prophylaxis (PEP) in exposed human cases.^1,6^ Cleaning the wound with water and soap is considered the first line of treatment and is necessary to remove viral deposits from the bite site, followed by seeking medical care immediately.^7^ Prompt administration of PEP minimises the frequency of fatalities by preventing the virus from spreading within the host.^8^

Despite the availability of safe and effective rabies vaccines and immunoglobulins to protect human life, rabies is still the cause of an estimated 59 000 human deaths and more than 3.7 million disability-adjusted life years per year globally.^8^ Although rabies vaccines are offered free of charge in South Africa, rabies outbreaks have been observed in the eThekwini Health district and surrounding areas.^9^ These occurrences of rabies infections have led to numerous fatalities among individuals, predominantly affecting children.^9^ While studies on the epidemiology of dog bites and human rabies have been conducted in KwaZulu-Natal province,^10,11,12^ to our knowledge, none have examined the knowledge, attitude and practices (KAP) of community members on dog bite management in the prevention of rabies in eThekwini District. This study aimed to assess the levels of KAP, along with associated factors regarding dog bite management for rabies prevention in the eThekwini District, KwaZulu-Natal province, in 2024.

## Research methods and design

### Study design

A cross-sectional community-based survey was conducted among household heads (HHs) from 06 May 2024 to 10 May 2024 in the South region of the eThekwini Metropolitan Health District in KwaZulu-Natal province, South Africa. This was a group research survey conducted by trainees and mentors participating in the South African Field Epidemiology Training Programme (SAFETP) – Intermediate training.

### Study setting

eThekwini district is located on the east coast of KwaZulu-Natal province, South Africa, and is the third largest metropolitan municipality in the country, following Johannesburg and Cape Town. The district comprises 103 wards that are urban, rural and peri-rural. The district includes three regions: the North, South and West. The district consists of one central hospital, five regional hospitals, 2 district hospitals, four specialised hospitals, eight community health centres (CHC) and 110 clinics, including 57 clinics under local authority.

### Study population

The heads of households from randomly selected households in the South region of the eThekwini district. The head of the household was defined as a person whom household members perceive to be the primary decision-maker in the family. In the absence of the head of the household, a responsible person above 18 years was interviewed. A household was excluded if the head of the household or, in their absence, the senior-most resident of the household was unavailable or did not have a person meeting the inclusion criteria or who declined to be interviewed.

### Sample size calculation

The sample size was calculated using Equation 1:^13^

$$n=\frac{z^{2}p(1-p)}{d^{2}}.$$ [Eqn 1]

Assuming the proportion of knowledge level of 50% because no similar study was conducted in the area with a 5% margin of error, we estimated a sample size of 384 at a 95% confidence level. We accounted for a 10% non-response rate, and the final sample size was determined to be 422.

### Sampling procedure

A stepwise approach was used to select study participants in the eThekwini district. Firstly, because of logistics and time constraints, a region that reported the highest number of dog bites and rabies cases in 2023 (South region) was purposively selected. Secondly, within the South region, we selected six (out of 73) public health facilities that reported the highest number of dog bite cases in 2023 (Kwa Makhutha CHC, Adams Clinic, Umbumbulu Clinic, Umlazi H and K clinics).

Finally, we obtained a complete list of villages under each public health facility catchment area (HFCA) to form a sampling frame. A health facility catchment area represents a geographical area around a health facility, describing most of the population using its services.^14^ We drew the required sample size from all the HFCAs using the probability proportional to size method. In selecting the households, a simple random sampling technique was used, employing a lottery method to ensure randomness to identify the starting point within the sample area. The randomised household was assigned the first household’s starting point. Every fifth household was interviewed. In every selected household, a consenting HH, or in their absence, the senior-most resident of the household who is over 18 years old, was purposively selected for interview. If the selected household had nobody present during the visit or did not have a person meeting the inclusion criteria, or if they declined to be interviewed, the next immediate household was visited.

### Data collection

A structured questionnaire was designed to collect the data from the participants based on a review of the literature. The questionnaire was written in English, translated into the local language (isiZulu) and retranslated into English by an independent translator to test for any differences or inconsistencies in the meaning of words and concepts. The questionnaire was pretested before the actual field survey in other districts of KwaZulu-Natal province.

Face-to-face interviews were conducted using a structured questionnaire. The field team consisted of 13 trainees enrolled in the intermediate training of the South Africa Field Epidemiology Training Programme, fluent in English and the local language (isiZulu). Data were collected using both Epi Info 7 mobile and paper-based questionnaires. The HH (or their representative) was interviewed for each household. The field team was trained to identify stress in the participants and was trained in counselling techniques, dealing with distress and a referral procedure.

### Data management and analysis

Data were entered into a database developed in Epi Info version 7.2.5.0 (CDC, Atlanta, Georgia, United States [US]).^15^ Data cleaning, management and analysis were done using Microsoft Excel (Microsoft Corp., Redmond, Washington, US)^16^ and STATA software version 16.^17^

A participant’s KAP regarding dog bite management in the prevention of rabies were the dependent variables in the analyses. In contrast, ‘age’, ‘gender’, ‘marital status’, ‘educational level’ and ‘dog ownership’ were the explanatory variables. Each individual’s total knowledge, attitude and practice scores were computed by adding their scores for each question. Bloom’s cut-off points were adapted and modified, wherein the total score obtained was converted to percentages.^3,18^ If all answers were complete and accurate, a respondent obtained an overall score of 22 for knowledge, 24 for attitudes and 15 for practices.^18^

The participants’ knowledge scores regarding rabies were determined as follows: The response to each correct knowledge question was scored 1, while a wrong or unsure response was scored 0. A total knowledge score was computed for each respondent out of a maximum score of 22 and converted to percentages. A ≥ 60% score of correct responses was assigned as good knowledge of dog bite management in rabies prevention. A total score of < 60% was classified as poor knowledge.^3,18^

Attitude was assessed using Likert’s scaling technique. The questions on Likert’s scaling had positive and negative responses ranging from ‘agree’ (score 3), ‘not sure’ (score 2) and ‘disagree’ (score 1). The responses were summed up, and a total score was obtained for each respondent out of a maximum score of 24 and converted to percentages. A score of ≥ 60% of correct responses was assigned as a positive attitude. A total score of < 60% was classified as a negative attitude toward dog bite management in the prevention of rabies.^3,18^

The participants’ practices were also determined using Likert’s scaling method. The scoring system of Likert-type scales for respondent’s responses ranging from ‘yes’ (score 3), ‘not sure’ (score 2) and ‘no’ (score 1) was used. The responses were summed up, and a total score was obtained for each respondent out of a maximum score of 15. A score of ≥ 60% of correct responses was assigned as good practices, while a total score of < 60% was classified as poor practices towards dog bite management in the prevention of rabies.^3,18^

Continuous variables were described by mean and standard deviation (s.d.), and categorical data were described using frequency and percentage. Univariable analyses were performed using the Chi-squared test of independence or Fisher’s exact test. Variables with a *p*-value ≤ 0.25 were then taken to a multivariable logistic regression model, and a final model was generated using a stepwise process to delineate factors independently associated with each outcome of KAP. Variables with a *p* < 0.05 were retained in the final model. The final model was evaluated with the Hosmer-Lemeshow goodness-of-fit test.^19^ Odds ratios (ORs) and 95% confidence intervals (CIs) were obtained from the final model.^20^

### Ethical considerations

Ethical clearance to conduct this study was obtained from the University of Pretoria Faculty of Health Sciences Research Ethics Committee (No. 120/2024) and the KwaZulu-Natal Department of Health and eThekwini District authorities. Before administering questionnaires, participants were informed about the purpose of the study using a standardised participant information sheet. Participants were asked to give written consent. No personal details of the participants, including their names, were recorded.

## Results

### Demographic characteristics of household heads

A total of 437 HHs were interviewed with a mean (s.d.) age of 40.6 ± 15.7 years. Most were females, 59% (*n* = 258/437) and 62% (*n* = 269/436) had secondary education and 46% (*n* = 220/430) were unemployed (Table 1).

**TABLE 1:** Socio-demographic characteristics.

Variable	*n*	%
**Age group (years)**		
18–30	134	30.7
31–50	200	45.8
≥ 51	103	23.6
**Sex**		
Male	179	41.0
Female	258	59.0
**Facility catchment area**		
Adams Clinic	86	19.7
KwaMakhutha Clinic	203	46.4
Umbumbulu Clinic	120	27.5
Umlazi H	11	2.5
Umlazi K	17	3.9
**Religion**		
Indigenous African	83	18.9
Christian	349	79.9
Muslim	5	1.1
**Level of education**		
No formal education	22	5.0
Primary	43	9.8
Secondary	269	61.6
Tertiary	103	23.6
**Marital status**		
Married	80	18.3
Single	330	75.5
Divorced	10	2.3
Widowed	17	3.9
**Occupation**		
Unemployed	241	55.1
Employed	132	30.2
Pensioner	37	8.5
Student	27	6.2
**Dog ownership**		
Yes	134	30.6
No	303	69.4
**Have you or any member of your family ever been bitten by a dog?**		
Yes	167	38.2
No	270	61.3

### Knowledge of dog bites management

Most (93.4%) participants said they had heard of rabies. Most (49.3%) of the participants indicated they heard about the disease from mass media (radio, television). Almost all participants (96.3%) identified dogs as the primary reservoir of rabies. Fewer participants could identify the main clinical signs of rabies in humans. However, hypersalivation (18.5%), barking (17.2%), hallucination (16.7%) and aggression (18.8%) were less commonly identified; participants recognised this as a sign in humans. More than half of the participants (68.1%) identified a bite from a rabid dog as the primary way that people acquire rabies. Additionally, 77.6% knew animal rabies vaccinations were free through government offices. The majority, 94% (95% CI: 91.4% – 95.9%), of participants had poor knowledge of dog bite management in the prevention of rabies (Table 2).

**TABLE 2:** Knowledge of participants towards dog bite management.

Variable	*n*	%
**Have you heard about rabies?**		
Yes	408	93.4
No	29	6.6
**What is your source of information? (*n* = 408)**		
Mass media (radio, television)	201	49.3
Social media	47	11.5
Government vaccination campaigns	142	34.8
**What is the cause of rabies?**		
Virus	260	59.5
Insufficient intake of food	21	4.8
Psychological problem	7	1.6
Chemical substances	7	1.6
Do not know	142	32.5
**What is the main reservoir/source of rabies?**		
Dog	421	96.3
Cat	175	40.1
Bat	14	3.2
**What is the mode of transmission of rabies from rabid animals to humans?**		
Bite	297	68.1
Saliva contact with open wound	106	24.3
Scratching	74	16.9
**What are the clinical signs and symptoms of rabies in humans?**		
Paralysis	29	6.6
Hypersalivation	81	18.5
Hydrophobia	18	4.1
Barking	75	17.2
Hallucination	73	16.7
Aggression	82	18.8
Difficulty swallowing	27	6.2
**Can rabies be prevented by vaccination of pets?**		
Yes	411	94.1
No	11	2.5
I do not know	15	3.4
**Which groups of people are at higher risk of rabies?**		
Children	369	84.4
Adults	181	41.4
**Which sex is at higher risk of rabies?**		
Male	310	71.0
Female	243	55.6
**Should dog bite wound be washed with soap and water before seeking medical attention?**		
Yes	221	50.6
No	131	29.9
I do not know	85	19.5
**Is vaccination of animals against rabies free of charge from authorised government offices?**		
Yes	339	77.6
No	75	17.2
I do not know	23	5.3
**Overall knowledge**		
Poor	411	94.1
Good	26	5.9

### Attitude towards dog bite management

Most (92.7%) participants agree that stray dogs are dangerous and that dog bites are the primary source of rabies transmission to humans (94.1%). Immediate anti-rabies injection after a bite is supported by 96.1%, and 92.7% believe rabies can be prevented through dog vaccination. Considering the total attitude score, 99.3% (*n* = 434) (95% CI: 98% – 99.8%) of the participants had a positive attitude (Table 3).

**TABLE 3:** Attitudes of participants towards dog bite management.

Statement	Agree		Not sure		Disagree	
	*n*	%	*n*	%	*n*	%
Stray dogs are dangerous	405	92.7†	24	5.5	8	1.8
Dog bites are the primary source of rabies transmission to humans	415	94.1†	17	3.9	5	1.1
Anti-rabies injection should be received immediately after being bitten by a dog	420	96.1†	10	2.3	7	1.6
Rabies is prevented through vaccination of dogs	405	92.7†	25	5.7	7	1.6
Rabies can be prevented by vaccination of human beings	255	58.3†	82	18.8	100	22.9
Rabies can be prevented by educating people about the disease	404	92.4†	17	3.9	16	3.7
If bitten by a dog, I should consult a traditional healer	52	12.1	29	6.6	356	81.5†
I am willing to vaccinate my dog	387	88.6†	41	9.4	9	2.1

Note: Overall Attitude: Positive – *n* = 434, 99.3%; Negative – *n* = 3, 0.7%.

† , Scores assigned to ‘favourable’ responses and reflect attitudes consistent with rabies prevention.

**TABLE 4:** Practices of participants with a history of dog bite towards dog bite management (*N* = 167).

Question	Yes		Not sure		No	
	*n*	%	*n*	%	*n*	%
Do you or any of your family members touch dogs	67	40.1	27	18.5	73	43.5†
Do you or your family members wash their hands after touching the dog(s)? (*n* = 83)	54	65.1†	23	27.7	6	7.2
Did you seek immediate medical attention after being bitten by a dog? (*n* = 167)	147	88.0†	9	5.4	11	6.6
Did you wash the dog bite wound with soapy water? (*n* = 167)	147	88.0†	9	5.4	11	6.6
Do you keep your dog in a fenced place? (*n* = 67)	21	31.3†	7	10.5	30	58.2
Do you vaccinate your dog? (*n* = 67)	24	35.8†	15	41.8	28	22.4

Note: Prevention practice level: Good – *n* = 65, 38.9%; Poor – *n* = 102, 61.1%.

† , Scores assigned to ‘favourable’ responses and reflect practices consistent with rabies prevention.

### Practices towards dog bite management

Among the 167 participants with a history of a dog bite, 88% reported having sought medical attention after being bitten by a dog. Of 67 who owned dogs, 35.8% reported that they always vaccinate their dogs. Only 31% of participants indicated that they keep their dogs in a fenced place. Approximately one-third, 38.9% (95% CI: 35.7% – 44.2%) of the participants have good practices for managing dog bites in preventing rabies.

### Factors associated with knowledge towards dog bite management

In the multivariable model, age and education were the only variables associated with good knowledge of dog bite management. The odds of good knowledge of dog bite management were 4.9 (*p* < 0.03; CI: 0.86–19.3) higher in the 31–50 years age group than in the 51 years and older group. Participants with secondary education had 61% (*p* < 0.03; CI: 0.17–0.92) lower odds of good knowledge than those with tertiary education (Table 5).

**TABLE 5:** Factors associated with good knowledge towards dog bite management.

Variable	Univariate			Multivariable		
	Crude OR	95% CI	*p*-value	aOR	95% CI	*p*-value
**Age group (years)**						
18–30	2.4	0.47–11.98	0.298	1.9	0.35–10.47	0.458
31–50	4.9	1.13–21.96	0.033	4.1	0.86–19.3	0.035
≥ 51	1.00	1.00	-	1.00	-	-
**Sex**						
Male	1.00	-	-	-	-	-
Female	1.1	0.49–2.52	0.789	-	-	-
**Religion**						
Christian	1.00	-	-	-	-	-
African	0.75	0.25–2.25	0.610	-	-	-
Muslim	0.00	0.00	-	-	-	-
**Level of education**						
No education	0.00	0.00	-	-	-	-
Primary	0.37	0.08–1.72	0.206	0.62	0.34–1.85	0.590
Secondary	0.35	0.15–0.82	0.015	0.39	0.17–0.92	0.031
Tertiary	1.00	-	-	1.00	-	-
**Marital status**						
Married	1.07	0.39–2.92	0.900	-	-	-
Unmarried	1.00	-	-	-	-	-
**Occupation**						
Employed	1.00	-	-	-	-	-
Unemployed	0.57	0.25–1.27	0.170	-	-	-
**Dog ownership**						
Yes	1.01	0.42–2.37	0.990	-	-	-
No	1.00	-	-	-	-	-
**Have you or any member of family ever bitten by a dog?**						
Yes	0.85	0.37–1.94	0.697	-	-	-
No	1.00	-	-	-	-	-

aOR, adjusted odds ratio; CI, confidence interval; OR, odds ratio.

### Factors associated with good practices towards dog bite management

The odds of practising good dog bite management were 17 times (aOR = 17.51; 95% CI: 10.3–29.6) higher among those who own a dog than those who do not own a dog (Table 6).

**TABLE 6:** Factors associated with good practices towards dog bite management.

Variable	Univariate			Multivariable		
	Crude OR	95% CI	*p*-value	aOR	95% CI	*p*-value
**Age group (years)**						
18–30	0.75	0.42–1.32	0.319	-	-	-
31–50	0.95	0.57–1.59	0.854	-	-	-
≥ 51	1.00	-	-	-	-	-
**Sex**						
Male	1.00	-	-	-	-	-
Female	0.84	0.55–1.28	0.425	-	-	-
**Religion**						
Christian	1.00	-	-	-	-	-
African	0.89	0.52–1.52	0.670	-	-	-
Muslim	1.55	0.25–9.41	0.634	-	-	-
**Level of education**						
No education	1.19	0.44–3.21	0.731	-	-	-
Primary	1.23	0.57–2.66	0.595	-	-	-
Secondary	1.08	0.65–1.78	0.764	-	-	-
Tertiary	1.00	-	-	-	-	-
**Marital status**						
Married	1.01	0.59–1.72	0.957	-	-	-
Unmarried	1.00	-	-	-	-	-
**Occupation**						
Employed	1.00	-	-	-	-	-
Unemployed	0.91	0.58–1.42	0.693	-	-	-
**Dog ownership**						
Yes	17.42	10.50–28.9	< 0.001	17.51	10.3–29.6	< 0.001
No	1.00	-	-	-	-	-
**Have you or any member of family ever bitten by a dog?**						
Yes	2.01	1.32–3.05	0.001	-	-	-
No	1.00	1.00	-	-	-	-
**Knowledge level**						
Good	1.05	0.44–2.48	0.907	-	-	-
Poor	1.00	1.00	-	-	-	-
**Attitude level**						
Positive	0.21	0.02–2.33	0.203	-	-	-
Negative	1.00	-	-	-	-	-

aOR, adjusted odds ratio; CI, confidence interval; OR, odds ratio.

## Discussion

This study investigated the factors linked to knowledge, attitudes and practices concerning dog bite management in the prevention of rabies among HHs in the South region of the eThekwini district, South Africa. The participants of the present study had positive attitudes (99.3%); however, they had a poor level of knowledge (94.1%) and practices (70.2%) according to the adopted scoring system.

Dogs are mainly responsible for transmitting rabies to humans.^1^ This is well-reported and known worldwide, including in South Africa.^9^ This study found that over 95% of the participants were aware that rabies is mainly transmitted by dogs. This is in agreement with the World Health Organization (WHO) report that 99% of rabies in humans is from rabid dog bites.^1^ Similar findings were reported in Bangladesh, Pakistan and Sri Lanka.^21,22,23^ In contrast, a study in Bangladesh^24^ reported a lower proportion (52.8%) of respondents who knew that rabies could be transmitted from dogs to humans. This variation might be associated with a difference in community awareness between different study areas.

Cleaning the dog bite wound with soap and water before hospital presentation is an important measure recommended by the WHO.^25^ Wound washing and flushing reduces the impact of the disease by fivefolds.^26^ Our results showed that half of the participants in this study were not aware of this preventive practice. Similarly, a study in Morocco reported that 56% of participants were unaware of the nonspecific treatment of washing the wound with soap and water.^27^ However, a study conducted in Ethiopia^28^ reported a higher proportion (70%) of participants who knew that a wound should be washed with soap and water as a first aid measure after a suspected animal bite. Lack of wound washing is responsible for a fivefold increase in the risk of developing rabies.^29^

The majority of participants had insufficient knowledge of the mode of transmission of rabies from an animal. Studies in Ethiopia and Bangladesh observed a similar knowledge gap among community members.^5,30^ More than half of the participants were unaware that rabies could be transmitted to humans through scratch and/or saliva contact with an open wound in addition to bites. Overall, our study found that 94% of participants had low levels of knowledge towards dog bite management in the prevention of rabies. This finding aligns with studies conducted in Maharashtra and Zimbabwe,^31^ where study participants showed low levels of knowledge.^31,32^

Understanding the community attitude and perceptions of treatment-seeking behaviours is important for rabies prevention in humans. In the current study, 99.3% of participants had a positive attitude towards dog bite management to prevent rabies. Similarly, a study in Ethiopia reported positive attitudes in 98.6% of participants.^33^ The findings are much higher compared to a previous study where only 56.2% of the participants had a positive attitude towards dog bite management in the prevention of rabies.^34^ Furthermore, other studies in Maharashtra and Bangladesh have reported low levels of positive attitude.^5,31^ More than 90% of participants agreed that anti-rabies injections should be received immediately after being bitten by a dog. This is similar to a study done in the Shinyanga region, Tanzania.^35^

The findings of this study indicate that only 29.8% of participants had good practice towards dog bite management. This is similar to the findings in the study done by Mahajan et al.^31^ wherein 13% of participants had good practices. This is lower than previous reports of 61.3% in Ethiopia and 74% in Nigeria.^33,36^ The variation in the proportion of appropriate practices for the compared studies may be because of the difference in the methodology used in scoring positive practices.

In this study, being 31–50 years old was associated with having good knowledge. Our study showed that participants who own a dog are more likely to have a good practice score than those who have no dog. A similar report from India indicates dog ownership has a positive contribution in performing preventive practice measures.^37^ This might be attributed to the expected risks associated with owning a dog.

The study had some limitations; it was limited to the South region, and the findings may not be generalisable to KwaZulu-Natal province and South Africa as a whole, but they did provide data for the eThekwini district. The sample of people interviewed from the household was those found at home during the visit; that is, Monday to Thursday (06–10 May 2024) between 8:00 and 17:00. Since women (particularly dependents/housewives) are more commonly present at home than men, the number of female respondents in this survey was more than that of males (59% vs. 41%). The reliance on self-reported data may introduce response bias, as participants might underreport negative practices or overstate their knowledge. Lastly, only adults were interviewed: those younger than 18 years of age were excluded because of ethical issues. We acknowledge that those who were not interviewed may have different knowledge, attitudes and practices of dog bite management. Given the above issues, the study results should be interpreted with a degree of caution.

## Conclusion

Despite a positive attitude towards dog bite management in rabies prevention, the knowledge and practices were poor in the eThekwini Health District, South Africa. The findings reveal significant gaps in knowledge and practice in the community. It is recommended that the government intensify awareness campaigns focused on dog bite management as a preventive measure against rabies.
